# Diagnosis of Peritoneal Tuberculosis from Primary Peritoneal Cancer

**DOI:** 10.3390/ijerph181910407

**Published:** 2021-10-03

**Authors:** I-Hui Chen, Pao-Ling Torng, Chia-Yi Lee, Kuang-Han Lee, Heng-Cheng Hsu, Wen-Fang Cheng

**Affiliations:** 1 Department of Obstetrics and Gynecology, National Taiwan University Hospital Hsin-Chu Branch, Hsin-Chu 300, Taiwan; dominique87@hotmail.com (I.-H.C.); plzfixthecar@gmail.com (C.-Y.L.); kuanghan.lee@gmail.com (K.-H.L.); b101092037@gmail.com (H.-C.H.); 2 Department of Obstetrics and Gynecology, National Taiwan University Hospital, National Taiwan University College of Medicine, Taipei 100, Taiwan; wenfangcheng@yahoo.com; 3 Graduate Institute of Clinical Medicine, College of Medicine, National Taiwan University, Taipei 100, Taiwan; 4 Department of Obstetrics and Gynecology, Chu-Tung Branch, Hsin-Chu 310, Taiwan; 5 Graduate Institute of Oncology, College of Medicine, National Taiwan University, Taipei 100, Taiwan

**Keywords:** tuberculous peritonitis, peritoneal neoplasms, symptom assessment, radiography

## Abstract

Peritoneal tuberculosis (PTB) is an uncommon extrapulmonary infection mimickng primary peritoneal cancer (PPC). We retrospectively included 23 women with PTB and 47 women with PPC treated in a medical center to study the clinical and radiological features that differentiate PTB from PPC. Body temperature above 38 °C was a unique feature of PTB (34.7% versus 0%, *p* < 0.001). Body Mass Index (BMI) was lower (21.9 ± 3.7 versus 25.2 ± 4.1, *p* = 0.003), white blood cell (WBC) count was lower (5179.6 ± 1502.2 versus 7716.2 ± 2741.8, *p* < 0.001), and CA-125 level was lower (508.0 ± 266.1 versus 2130.1 ± 2367.2 U/mL, *p* < 0.001) in PTB compared with PPC. Imaging detected more pulmonary infiltration and consolidation (52.2% versus 6.4%, *p* < 0.001), and less omental/mesentery changes (52% versus 83%, *p* < 0.001) in PTB compared with PPC. The operated patients received earlier treatment compared to patients without operation (7.9 ± 5.3 days versus 17.2 ± 11.0 days, *p* = 0.010). In conclusion, fever above 38 °C, lower BMI, lower WBC count, less elevated CA-125 level, and imaging of less omental involvement were features of PTB differentiated from PPC.

## 1. Introduction

Tuberculosis (TB) is an important global health problem. According to the World Health Organization, it was one of the top 10 causes of death worldwide in 2018 and is the leading cause of death of people with human immunodeficiency virus (HIV) [1]. Globally, an estimated 1.3 million people died from TB each year and an estimated 10.0 million people newly sick with TB in 2018 [2]. More than half of TB were diagnosed in developing countries, including India, Indonesia, China, the Philippines, and Pakistan [2]. In Taiwan, TB is decreasing year by year. Yet, there were 9759 new cases and 511 TB-related deaths in 2017 [3], suggesting that TB is still an important transmissible disease in Taiwan.

Peritoneal tuberculosis (PTB) is an uncommon form of extrapulmonary tuberculous infection. Several possible pathogeneses of PTB have been reported, such as hematogenous spread of pathogen from a primary pulmonary infection focus, rupture of caseous abdominal lymph nodes, direct spread from focus in the intestine or fallopian tubes, and direct contamination in patients who receive peritoneal dialysis [4]. Clinical symptoms for PTB are often non-specific, including abdominal distension, abdominal pain and body weight loss [5]. These clinical presentations of PTB showed high similarities to advanced epithelial ovarian cancer and disease with peritoneal pathologies, especially the primary peritoneal cancer (PPC) [5,6,7,8,9]. 

In imaging studies such as computed tomography (CT), three patterns of peritoneal involvement in PTB have been described: wet type characterized by large amounts of ascites (90%); fibrotic-fixed type characterized by matted bowel loops and mesentery, omental mass with small volume of ascites (7%); and dry type characterized by dense adhesions, fibrous peritoneal reaction, and caseous nodules (3%) [10]. These image characteristics also mimic PPC and lead to misdiagnosis [11].

Primary peritoneal cancer (PPC) is an uncommon peritoneal neoplasm. Like serous primary ovarian cancer, its precursor lesion may be serous tubal intraepithelial carcinomas (STIC) [12]. PPC usually arises multifocally with rare or only superficial involvement to ovaries, and has a poor overall survival as in advanced epithelial ovarian cancer [12]. In contrast, PTB is a medically curable disease with good survival rate.

In this study, we analyzed the clinical features of PTB and PPC, including symptoms and signs, level of tumor markers and characteristics in imaging studies. We aim to identify factors that could differentiate these two diseases to make an early and more effective diagnosis of PTB for earlier treatment.

## 2. Materials and Methods

This study was conducted with approval from the institutional review board at The National Taiwan University College of Medicine (201112066RIC). Medical records were reviewed to identify patients diagnosed with PTB between January 2006 and March 2018. PTB was diagnosed based on final pathologic report, or Mycobacterium tuberculosis growth or tuberculosis Polymerase chain reaction (PCR) test in ascites or lymph nodes. Patients who were diagnosed as PPC during the same time period were included for comparison. The diagnosis of PPC was made based on histopathology according to the Gynecologic Oncology Group criteria as following: (1) both sides of the ovaries must be in a normal size or enlarged due to benign processes; (2) extent of involvement must be more at the extra-ovarian than either of the ovarian surface; (3) the microscopic nature of the ovarian component must be: (a) non-existent, (b) confined only to the surface ovarian epithelium with no invasion to the cortex, (c) with involvement of the surface of ovarian epithelium and underlying stromal of cortex with any tumor size less than 5 × 5 mm [12].

Clinical data, including patient’s age at diagnosis, medical illness, symptoms, and results of physical examinations and laboratory data, including hemogram, white blood cell (WBC) count, and preoperative cancer antigen 125 (CA-125) values were recorded. Fever was defined as body temperature higher than 38 degrees Celsius. Imaging studies including chest X-ray and computed tomography (CT) of the abdomen and pelvis were reviewed. Details of the CT, such as the extent of omental or mesenteric involvement and peritoneal involvement, the amount of ascites, and the presence of lymphadenopathy, were classified according to the criteria proposed by Choi et al. [13]. The degree of omental or mesenteric involvement was graded as none, mild (infiltration was limited to the omentum and the mesentery), or severe (masses were detected). The amount of ascites was classified as none, small (in either the abdomen or the pelvis), moderate (in both the abdomen and the pelvis, but the abdomen was not distended), or large (in both the abdomen and the pelvis with abdominal distension). Peritoneal involvement was graded as none, smooth thickening, or nodular thickening. 

Patients with PTB were admitted to different departments due to the insidious initial presentations. Requirement for surgery were judged by in-charged physicians based on clinical conditions. Surgery either by laparoscopy or laparotomy was performed by either gynecologists or general surgeons. All patients with PPC underwent cytoreductive surgery by gynecologic oncologists before initiation of chemotherapy or other treatment. In the PTB group, the time duration between initial patient admissions to anti-tuberculosis therapies were also recorded. 

Continuous variables were compared using the Mann–Whitney test. Nonparametric dichotomous variables were compared using an χ^2^ test or Fisher’s exact test. The statistical analysis was carried out using Statistical Package for the Social Sciences (SPSS) version 20.0 for Windows (SPSS Inc., Chicago, IL, USA). Continuous variables are reported as mean and standard deviation, while discrete variables are reported as percentages of the total. Probability values less than 0.05 were regarded as significant.

## 3. Results

### 3.1. Patient Characteristics

In total, 23 patients with PTB and 47 patients with PPC were included. Table 1 shows the clinical characteristics of these two groups of patients. The patients of PTB were in a wide range of age, but the mean age was similar between the two groups of patients. There were no differences in underlying medical diseases such as: cardiovascular disease, diabetes mellitus, thyroid disease, depression, stroke, and systemic lupus erythematous. However, patients with PTB have significantly lower BMI than patients with PPC. Presenting symptoms such as abdominal distention, abdominal pain, cough, poor appetite, or history of body weight loss were of no difference between the two groups. However, patients with PTB had significantly higher body temperature than patients with PPC. Eight (35%) patients in the PTB group, while none of the patients in PPC, presented with fever. In laboratory tests, patient with PPC showed significantly lower WBC count and lower CA-125 level.

### 3.2. Radiologic Characteristics

The radiologic characteristics are listed in Table 2. From chest X-rays, patients with PTB had more pulmonary infiltration or consolidation than patients with PPC. In the CT images, there were no differences in the amount of ascites and peritoneal thickening in both groups. However, patients of PPC had significantly more prominent omental and mesentery changes than patients of PTB. Patients with PPC had more large lymphadenopathy than those with PTB (10.6% versus 0%).

### 3.3. Intraoperative Findings

**Figure 1:**
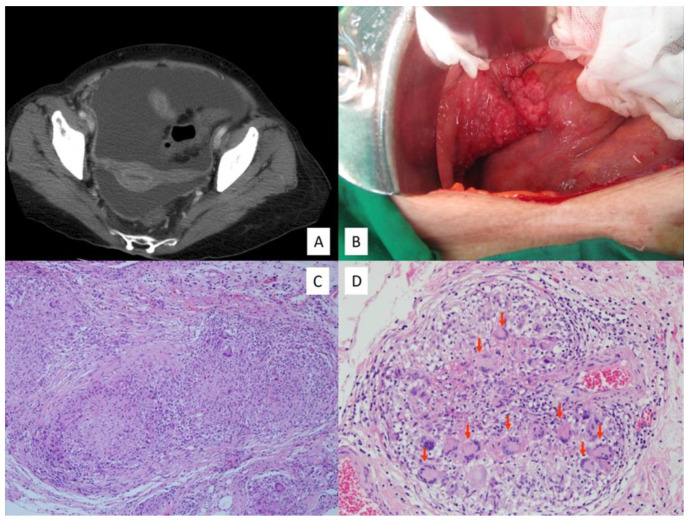
(**A**) Pelvic computed tomography of peritoneal tuberculosis showed massive ascites with thickened peritoneum. (**B**) Intra-operative findings show multiple miliary nodules over the fallopian tube, bowel serosa, and parietal peritoneum. (**C**) Histology from peritoneal tuberculosis showed granulomas with caseous necrosis and (**D**) multinuclear giant cells (red arrows) in the granuloma (Hematoxylin and Eosin stain).

In the PTB group, 16 patients (69.6%) underwent operation either by laparoscopy (66.7%) or laparotomy (33.3%). Table 3 shows the intraoperative findings between the two groups of patients. Consistent with the CT image findings (Figure 1A), there were no differences in the amount of ascites and the peritoneal thickening in the two groups of patients. Microscopically, the surgical specimens in all of the PTB patients, except one case whose lesions revealed only chronic inflammation, showed miliary whitish nodules over the whole peritoneal surface of the genital organs (Figure 1B). PTB was diagnosed through typical peritoneal pathological findings of multiple granulomas with epithelioid cells and caseous necrosis (Figure 1C), and multinuclear giant cells (Figure 1D) [14].

### 3.4. Duration before Anti-Tuberculosis Therapy in PTB Patients

We calculated the duration of hospital stay from patient workup after admission to the initiation of anti-tuberculosis therapy in the PTB patients. In patients without surgery, PTB was diagnosed from ascites tapering for mycobacterial culture or tuberculosis PCR test. The duration was significantly shorter in patients with surgery versus those without surgery (7.9 ± 5.3, range 3–18 days versus 17.2 ± 11.0, range 6–34 days, *p* = 0.010). There was no difference in age, BMI, and CA-125 levels between the patients with surgery versus those without surgery.

## 4. Discussion

Our study shows that patients with PTB were diagnosed at a wider range of age, with lower BMI, higher rate of fever and more leukocytosis than patients with PPC. These results agree with the insidious nature of PTB and the chronic infectious illness of this disease.

In addition, patients with PTB had elevated serum CA-125 levels while those with PPC showed even higher CA-125 levels. The medium CA-125 levels were reported as 448 to 508 in patients with PTB and 1484 to 2626 in patients with PPC [13,15,16], suggesting that CA-125 in PPC were 3 to 5 times higher than in PTB. In addition to CA-125, CA 19–9 and carcino-embryonic antigen (CEA) [17] were also serum markers that were elevated in patients with PPC, and could be used to differentiate PTB from PPC. 

Patients with PTB usually present with much ascites that mimic PPC [5,7,8,9]. In our study, we also found the amount of ascites from imaging studies (Table 2) and from operation records (Table 3) indistinguishable between these two groups of patients. Paracentesis is usually performed to obtain ascites fluid for disease diagnosis. However, the accuracy of various pre-operative diagnostic tests for PTB using ascites was limited. It was reported that the detection rate of finding ascites such as predominant lymphocytes, LDH, AFS, and mycobacterial culture were only 68%, 77%, 3%, and 35%, respectively, for PTB. [14] Polymerase chain reaction (PCR), a method for detecting Mycobacterium tuberculosis also shows various ranges of sensitivity [14,18]. Some studies reported that the ascitic adenosine deaminase (ADA), a purine-degrading enzyme that is 10–12 times higher in T cells than in B cells, could be a valuable marker for PTB diagnosis [19,20,21]. However, this test was not yet a standard procedure for PTB diagnosis. The pathological findings such as granulomas, characterized by Langhans type giant cells and caseous necrosis are typical of PTB [22]. Therefore, peritoneal nodular lesions biopsy achieved by surgery remains the most definite way to confirm the diagnosis. Our study shows that the duration from initial admission to anti-tuberculosis treatment after confirmation of PTB is much longer in patients without operation than patients receiving operation (mean 17.2 versus 7.9 days). Although PTB is a medically curable disease with good survival rate, longer duration between showing symptoms and receiving treatment increases mortality rate. Hence, early diagnosis with immediate anti-tuberculosis treatment would improve the prognosis [23]. Therefore, it is vital to identify patients with PTB to perform operations for an earlier diagnosis of PTB and its treatment.

Chest X-rays are routine image studies in hospitalized patients with illness. We detected more prominent pulmonary infiltration and consolidation in patients with PTB than in patients with PPC, suggesting that the lungs might be the primary origin of tuberculosis. However, such difference could be useless and non-specific to diagnose pulmonary tuberculosis and to differentiate PTB from PPC.

In terms of computed tomography (CT) imaging, we found that peritoneal thickening and detection of pelvic mass were also non-specific to distinguish PPC from PTB. Other studies also support this observation [24]. What does differentiate the two is that PPC has a significantly higher percentage of severe/mass-like omental and mesentery changes (74% versus. 4%, *p* < 0.001) compared with PTB. Additionally, images with large lymphadenopathy were specific to PPC than PTB (10.6% versus 0%). These findings were quite consistent with the literature reports listed in Table 4. 

**Figure 2:**
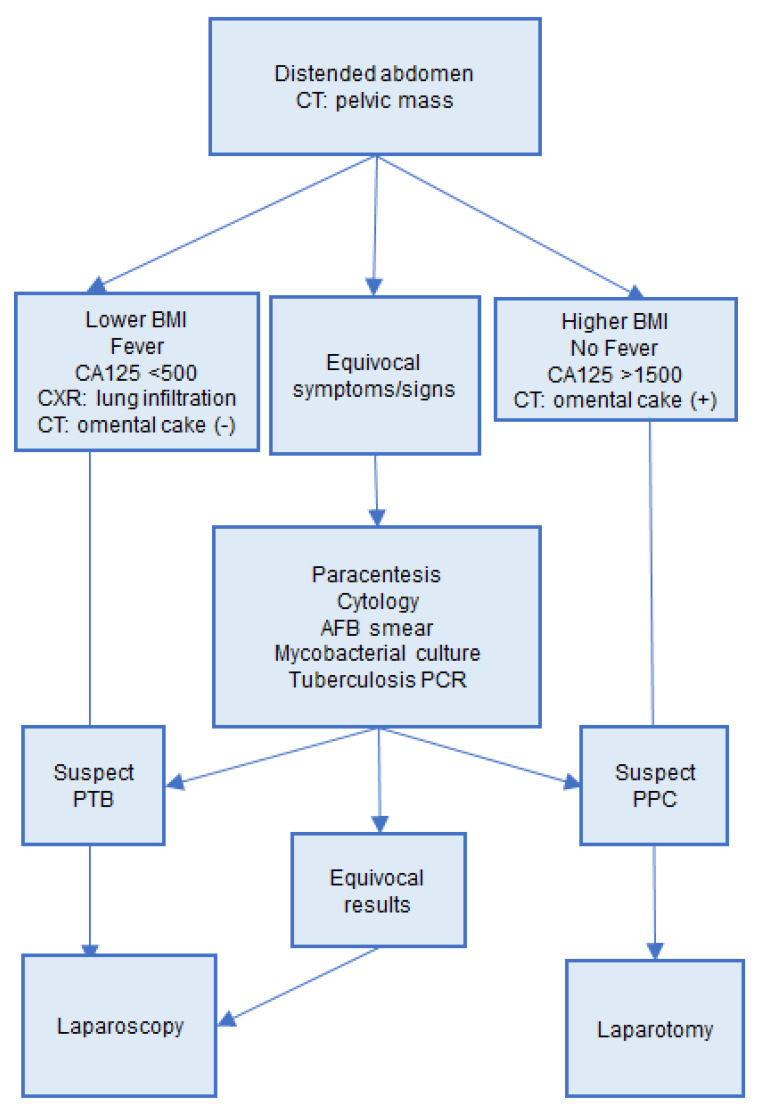
Algorithm for differential diagnosis of PTB and PPC. PTB, peritoneal tuberculosis; PPC, primary peritoneal cancer.

Laparoscopy has been believed to be the gold standard for the diagnosis of PTB with impressive sensitivity and specificity [27]. Our study confirms that laparoscopy gives a precise diagnosis, speeds up the diagnosis duration, and provides an earlier treatment. In our study, time duration was shortened by nine days in our patients who received surgery compared to those who did not have surgery. However, only 10 patients in our PTB group received laparoscopy. The five patients who received laparotomy and the eight patients who did not have operation could be due to inadequate interpretation of the patients’ conditions. Here, we propose an algorithm to help physicians to raise suspicions for PTB (Figure 2). PTB is suspected in patients with fever at above 38 °C, pulmonary infiltration upon CXR, no obvious omental cake in CT study, and lower levels of CA125. In these patients, we recommend laparoscopic biopsy for an earlier diagnosis of PTB and anti-tuberculosis treatment. On the other hand, PPC were more likely in patients with no fever, no pulmonary infiltration upon CXR, omental cake by CT, and higher levels of CA125, and recommend proceeding to debulking surgery. 

## 5. Conclusions

There has been a lack of clinical tools to provide a fast and accurate diagnosis of PTB. We observed a specific clinical spectrum of patients with PTB. Patients with the following features should be suspected of the higher possibility of PTB: lower BMI, fever, a serum CA-125 level around 500 U/mL, a lower WBC count, CXR showing infiltrative lesions, and CT study that revealed no obvious omental nodules. In these patients, laparoscopic biopsy could provide fast and effective diagnosis and shorten the time interval from workup to ant-tuberculosis therapy. We propose an algorithm for better and faster differentiation of PTB and PPC. Those who are suspected with PTB should be diagnosed through laparoscopy, which provides faster diagnosis and a shorter symptom-to-treatment time. 

## Figures and Tables

**Table 1:** Clinical characteristics and laboratory data.

	PTB (*n* = 23)	PPC (*n* = 47)	*p* Value
Age, years, mean ± SD range	57.5 ± 16.9	61.5 ± 8.6	0.29
	(23–83)	(41–78)	
Underlying disease * n (%)	15	26	0.23
	(65.2)	(55.3)	
BMI, kg/m^2^, mean ± SD range	21.9 ± 3.7	25.2 ± 4.1	0.003
	(17.1–32.4)	(18.9–38.8)	
Presenting symptoms, n (%)			
Abdominal distension	15 (65.2)	33 (70.2)	0.44
Abdominal pain,	9 (39.1)	12 (25.5)	0.19
Cough	7 (30.4)	12 (25.5)	0.46
Poor appetite	8 (34.7)	17 (36.2)	0.56
Body weight loss	7 (30.4)	11 (23.4)	0.36
Body temperature	37.8 ± 1.3	36.7 ± 0.6	0.001
Fever, n (%)	8 (34.7)	0	<0.001
Laboratory data			
CA125, U/mL, mean ± SD	508.0 ± 266.1	2130.1 ± 2367.2	<0.001
WBC count, K/uL, mean ± SD	5179.6 ± 1502.2	7716.2 ± 2741.8	<0.001

PTB, peritoneal tuberculosis; PPC, primary peritoneal cancer. * Under lying disease: cardiovascular disease, diabetes mellitus, thyroid disease, depression, stroke, and systemic lupus erythematous. Fever: Body temperature > 38 °C.

**Table 2:** Radiographic characteristics.

	PTB (*n* = 23)	PPC (*n* = 47)	*p* Value
Chest X-ray			
Pleural effusion, n (%)	11 (47.8)	12(25.5)	0.11
Pulmonary infiltration/ consolidation, n (%)	12 (52.2)	3 (6.4)	<0.001
Computer Tomography			
Pelvic mass	5 (21.7)	13(27.7)	0.81
Ascites			0.51
None	2(8.7)	7 (14.9)	
Small	5 (21.7)	5 (10.6)	
Moderate	5 (21.7)	6 (12.8)	
Massive	11 (47.8)	29 (61.7)	
Omental and mesentery changes			<0.001
None	9 (39.1)	5 (10.6)	
Mild/nodular	11 (47.8)	4 (8.5)	
Severe/mass	1 (4.3)	35 (74.5)	
NA	2	3	
Peritoneum changes			0.67
None	5 (21.7)	7 (14.9)	
Thickening	11 (47.8)	28 (59.6)	
Nodular	5 (21.7)	12 (25.5)	
Lymphadenopathy			0.16
None/Small	23 (100)	42 (89.4)	
Large	0	5 (10.6)	

PTB, peritoneal tuberculosis; PPC, primary peritoneal cancer. NA: missing data because abdominal CT was not done.

**Table 3:** Intraoperative findings.

	PTB (*n* = 16)	PPC (*n* = 47)	*p* Value
Omentum			<0.001
None, n (%)	9 (52.9)	6 (12.7)	
Nodule, n (%)	3 (17.6)	20 (42.6)	
Cake, n (%)	0	16 (34.0)	
NA	5	5	
Peritoneum			0.96
None, n (%)	2 (11.8)	8 (17.0)	
Papule, n (%)	2 (11.8)	3 (6.4)	
Miliary seedings	8 (47.1)	26 (55.3)	
Nodule (>1 cm), n (%)	5 (29.4)	9 (19.1)	
NA	0	1	
Ascites, mL, mean ± SD	1368 ± 1462.5	1844.2 ± 2008.8	0.33

PTB, peritoneal tuberculosis; PPC, primary peritoneal cancer. NA: missing data, un-recorded.

**Table 4:** List of clinical presentations in PTB and PPC from literature reports.

		Laboratory	Symptoms/Signs			Radiographic Findings			
	Age, Year	CA-125 Level	Abdominal Distension	Abdominal Pain	Fever	Pleural Effusion	Severe Omentum Involvement	Peritoneal Thickening	Ascites (Large Amount)
Ha et al., 1996 (study period: 1991–1994) [25]									
PTB (n = 42)	37 (8–74)	NA	NA	NA	NA	NA	5%	NA	31%
PPC (n = 93)	51 (19–89)	NA	NA	NA	NA	NA	17%	NA	30%
Choi et al., 2010 (study period: 1996–2006) [13]									
PTB (n = 20)	39 (23–69)	448 (32–1725)	70%	50%	20%	20%	45%	35%	55%
PPC (n = 17)	63 (50–73)	1484 (42–14,380)	53%	24%	0	18%	76%	41%	41%
Wang et al., 2012 (study period: 1995–2010) [16]									
PTB (n = 30)	39 ± 17	345 (0.6–850)	53%	23%	NA	13%	NA	3%	67%
PPC (n = 38)	60 ± 11	2626 (35–>500)	79%	18%	NA	24%	NA	34%	76%
Koc et al., 2006 (study period: 1992–2004) [5]									
PTB (n = 22)	37 (21–68)	565 (3–2021)	82%	55%	9%	23%	9%	5%	100%
Lataifeh et al., 2014 (study period: 2002–2012) [9]									
PTB (n = 16)	30 (13–65)	319 (45–1072)	81%	81%	38%	25%	50%	6%	100%
Oge et al., 2012 (study period 2000–2011) [8]									
PTB (n = 20)	38 (16–70)	289 (4–793)	65%	70%	15%	NA	30%	65%	85%
Hong et al.; 2011 (study period 2002–2010) [15]									
PTB (n = 60)	49 (21–80)	474 (61–1251)	71%	37%	37%	29%	NA	NA	93%
Zissin et al., 2001, (study period 1988–1999) [26]									
PTB (n = 19)	48 (20–85)	NA	16%	16%	NA	5%	53%	42%	53%
Current Study (study period: 2006–2018)									
PTB (n = 23)	58 (23–83)	508 (82.3–1214)	65%	39%	48%	48%	4%	70%	48%
PPC (n = 47)	62 (41–78)	2130 (56.9–11751)	70%	26%	4.2%	26%	74%	85%	62%

PTB, peritoneal tuberculosis; PPC, primary peritoneal cancer. Data were presented as medium (range), mean ± SD, range, or as percentage.

## Data Availability

The data presented in this study are available on request from the corresponding author. The data are not publicly available due to ethical and privacy issues.
